# Panduratin A Inhibits the Growth of A549 Cells through Induction of Apoptosis and Inhibition of NF-KappaB Translocation

**DOI:** 10.3390/molecules16032583

**Published:** 2011-03-21

**Authors:** Shiau-Chuen Cheah, David R. Appleton, Sui-Ting Lee, May-Lynn Lam, A. Hamid A. Hadi, Mohd. Rais Mustafa

**Affiliations:** 1 Centre of Natural Products & Drug Discovery (CENAR), Department of Pharmacology, Faculty of Medicine, University of Malaya, 50603 Kuala Lumpur, Malaysia; E-Mails: drappleton@hotmail.com (D.R.A.); suiting87@siswa.um.edu.my (S.T.L.); rais@um.edu.my (M.R.M.); 2 Sime Darby Technology Centre, 1st Floor, Block B, UPM-MTDC Technology Centre III, UPM, Serdang 43400, Selangor, Malaysia; 3 Cancer Research Initiatives Foundation (CARIF), 2nd Floor, Outpatient Centre, Sime Darby Medical Centre, 47500 Subang Jaya, Selangor Darul Ehsan, Malaysia; E-Mail: lammaylynn@hotmail.com (M.-L.L.); 4 Centre of Natural Products & Drug Discovery (CENAR), Department of Chemistry, Faculty of Science, University of Malaya, 50603 Kuala Lumpur, Malaysia; E-Mail: ahamid@um.edu.my (A.H.A.H.)

**Keywords:** Panduratin A, apoptosis, High Content Screening, Real-time Cellular Analyzer, NF-κB

## Abstract

In the present study we investigated the effects of panduratin A, isolated from *Boesenbergia rotunda*, on proliferation and apoptosis in A549 human non-small cell lung cancer cells. Cell proliferation and induction of apoptosis was determined by the real-time cellular analyzer (RTCA), MTT assay and High Content Screening (HCS). The RTCA assay indicated that panduratin A exhibited cytotoxicity, with an IC_50_ value of 4.4 µg/mL (10.8 µM). Panduratin A arrested cancer cells labeled with bromodeoxyuridine (BrdU) and phospho-Histone H3 in the mitotic phase. The cytotoxic effects of panduratin A were found to be accompanied by a dose-dependent induction of apoptosis, as assessed by DNA condensation, nuclear morphology and intensity, cell permeability, mitochondrial mass/ potential, F-actin and cytochrome c. In addition, treatment with an apoptosis-inducing concentration of panduratin A resulted in significant inhibition of Nuclear Factor-kappa Beta (NF-κB) translocation from cytoplasm to nuclei activated by tumor necrosis factor-alpha (TNF-α), as illustrated by the HCS assay. Our study provides evidence for cell growth inhibition and induction of apoptosis by panduratin A in the A549 cell line, suggesting its therapeutic potential as an NF-κB inhibitor.

## 1. Introduction

**Figure 1:**
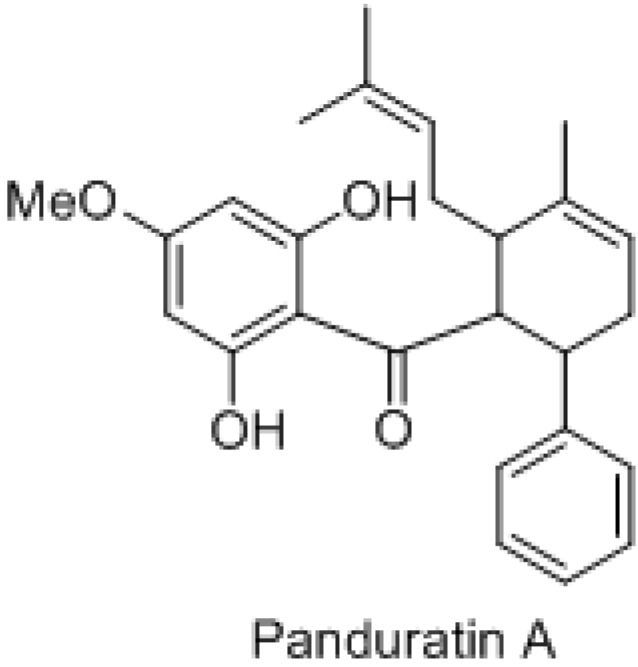
Chemical structure of panduratin A.

The rhizomes of *Boesenbergia rotunda* (fingerroot, Chinese ginger) have been used as a condiment and folk medicine for the treatment of various ailments, including colic disorders, fungal infections and muscular pains [1]. *In vitro* studies on the extracts of *B. rotunda* and their isolated compounds have shown some beneficial pharmacological activities. Panduratin A (CAS Registry Number: 89837-52-5; molecular weight for C_26_H_30_O_4_: 406.52; Figure 1), a cyclohexenyl chalcone derivative isolated from *B. rotunda*, showed strong antimutagen activity in the Ames test using *Salmonella typhimurium* TA98 [2]. Besides that Tuchinda *et al*. reported that panduratin A had significant antiinflammatory activity against 12-*O*-tetradecanoylphorbol 13-acetate (TPA)-induced ear edema in rats. Recently, Yun *et al*. reported that panduratin A inhibited the growth of HT-29 colon cancer cells and induced apoptosis [3]. Moreover, the same authors also reported that panduratin A induced apoptosis and cell cycle arrest in human PC3 prostate cancer cells and MCF-7 breast cancer cells [4]. However, to our knowledge no research had been done on A549 cells and the role of NF-kB in the cancer cell inhibition of panduratin A.

The majority of anticancer drugs presently used in clinical settings have been described to induce cell death by apoptosis [5]. Apoptosis has been the subject of intense study and was originally described by Kerr *et al*. [6] as cell death with certain morphological characteristics. However, besides the morphological criteria, subsequent studies have described important biochemical hallmarks of apoptosis. One of the most important groups of proteins involved in apoptosis is the nuclear factor-kappa B (NF-κB). NF-κB is a ubiquitous transcription factor which plays an important role in many physiological processes, such as cell proliferation, cell death, inflammation and immune response [7]. Under resting conditions, NF-κB is present as an inactive heterotrimer which consists of p50, p65, and I kappa B alpha (IκBα) subunits in the cytoplasm. Following activation by numerous of stimuli, IκBα protein undergoes phosphorylation and degradation. Unbound p50-p65 heterodimer translocates to the nucleus, subsequently binds with specific DNA motif in the promoter regions of target genes and activates their transcription. Dysregulation of NF-κB is implicated in many types of human cancers [8]. Thus, the inhibition of this signaling pathway could be able to halt tumor development.

Recently we isolated panduratin A from the methanolic extract of *B. rotunda* [2,3] and in the present study, we demonstrate antiproliferative and proapoptotic effect of this compound in human A549 non-small cell lung cancer cells and delineate the mechanism of this effect.

## 2. Results and Discussion

### 2.1. Results

#### 2.1.1. Panduratin A Isolation

*B. rotunda* has been shown [2,3] to contain the compound panduratin A (Figure 1), which was eluted with approximately 90% acetonitrile/water containing 0.1% (v/v) FA. Isolated panduratin A was shown to have an accurate mass of 407.2215 [(+)-ESI HRMS [M+H]^+^, ∆0.5 ppm for C_26_H_31_O_4_]. 

#### 2.1.2. Real-Time Cell Proliferation Assays

**Figure 2:**
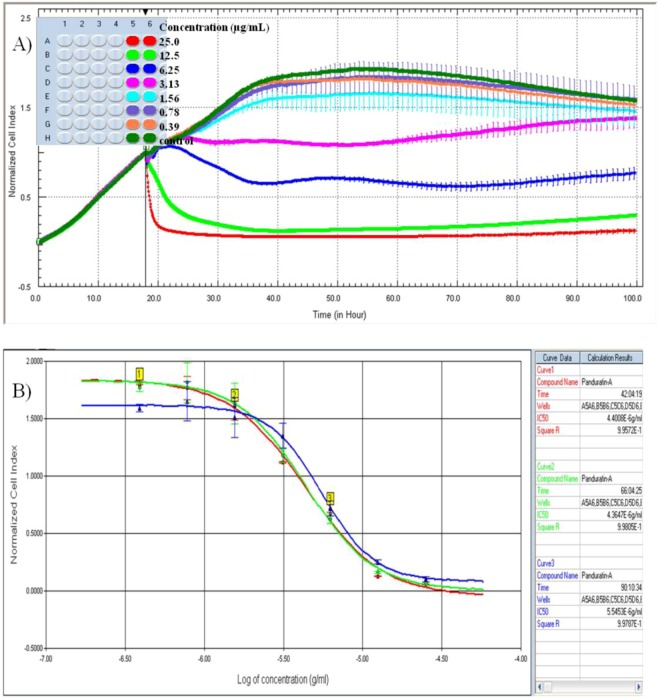
**(A)** Dynamic monitoring of drug interaction with target cells using the RTCA. A549 cells were seeded in 16X E-plate device were continuously monitored up to 100 hours at which point panduratin A were added at the indicated final concentrations. CI values were normalized to the time point of compound addition, indicated by the vertical black line. **(B)** IC_50_ value in RTCA system after 24, 48 and 72 hours treatment of panduratin A.

In order to observe the kinetics of drug interaction with target cells, A549 were seeded in an 16X E-plate™ and continuously monitored until the cells reached the log growth phase, at which point different concentrations of panduratin A were added to the cells at the indicated final concentrations. From Figure 2A, the compound, or medium only were added at the time recorded CI values reached one third of the maximum CI, approximately 18 hours after seeding, when cells were in early logarithmic growth phase. Changes in the CI values were recorded immediately with panduratin A treated A549 cells.

As shown in Figure 2A, a large decrease in CI was observed, reaching zero approximately 2 hours after treatment in higher dose (25 and 12.5 µg/mL of panduratin A), when cells had detached from the microelectrode surface. Panduratin A at the highest concentration initially induces a cytotoxic effect which is mainly due to cell death. Remarkably, the cells recover from the initial cytotoxic effect of the drug and start to re-proliferate in Figure 2A (doses of 3.13 and 6.25 µg/mL). All control cells, treated with medium alone, showed nearly identical CI profiles until the end of data collection, indicating similar growth behavior. Different curves observed immediately after panduratin A addition may reflect receptor and compound transportation events and need to be further evaluated. The sigmoidal dose response curves of panduratin A tested in RTCA are shown in Figure 2B. All curves have an r^2^ greater than or equal to 0.99.

#### 2.1.3. MTT Assay

**Figure 3:**
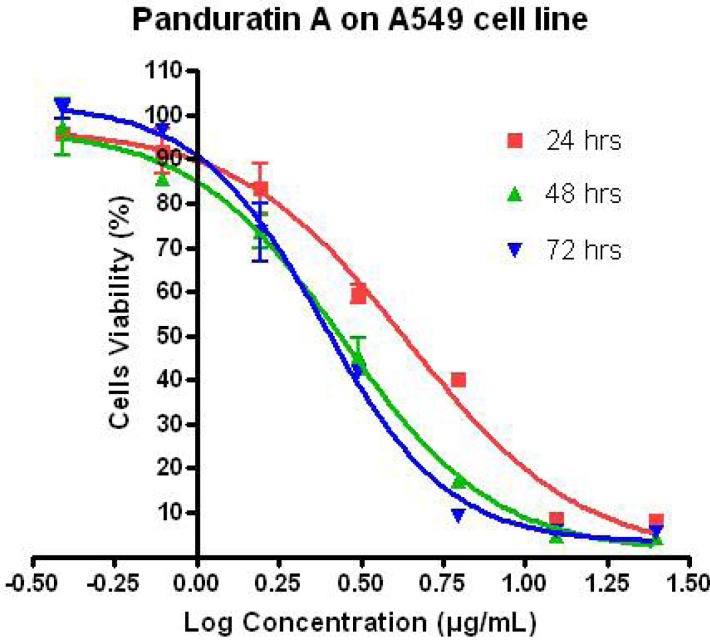
Dose response curves (using GraphPad Prism) of panduratin A tested in the MTT assays at 24, 48 and 72 hours treatment.

The sigmoidal dose response curves of panduratin A in the end-point assays are shown in Figure 3. All curves have an r^2^ greater than or equal to 0.95. Cell viability was analyzed using the MTT assay, measuring metabolic activity. In cells with normal metabolism, a tetrazolium salt is converted to formazan, measurable by change in absorbance. In A549 cells treated with panduratin A, metabolic activity decreased 24 hours after treatment. Medium alone experiment showed no effect on A549 cell viability and metabolism. Table 1 summarizes the IC_50_ values from RTCA (real-time assay) and MTT (end-point assay).

**Table 1:** IC_50_ values (µg/mL) at 24, 48 and 72 hours for panduratin A on A549 cells.

Panduratin A	RTCA	(µg/mL)	MTT	(µg/mL)
	IC_50_	SEM	IC_50_	SEM
**24 hrs**	4.4008	0.9782	4.4040	0.3460
**48 hrs**	4.3647	0.0728	3.7930	0.0156
**72 hrs **	5.5453	0.0066	4.4190	0.0062

#### 2.1.4. Cell Cycle Phases Determination

**Figure 4:**
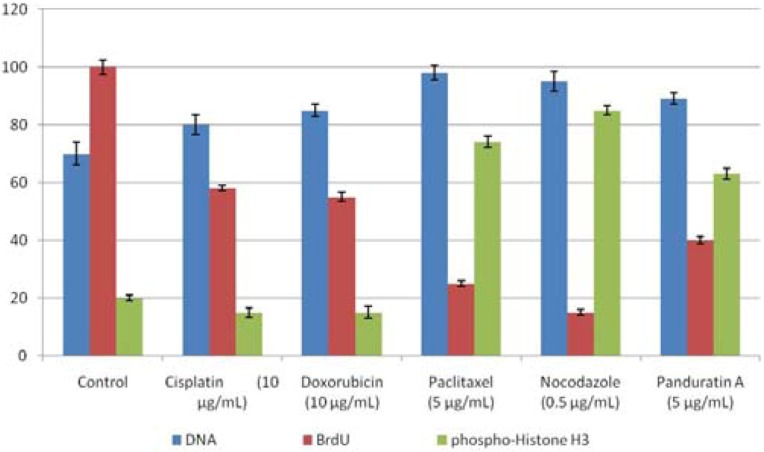
A549 cells were treated with medium or 5 µg/mL of cisplatin, doxorubicin, paclitaxel and panduratin A. Activation or inhibition of BrdU and hosphor-histone H3 after drug treatment for 24 hours being calculated and analyzed.

**Figure 5:**
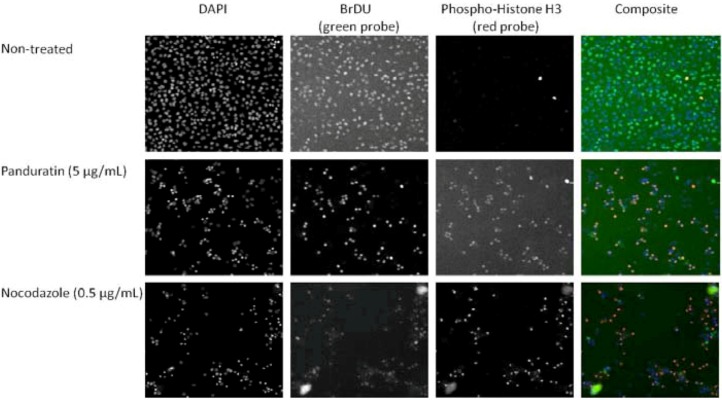
Staining of BrdU and hosphor-Histone H3 in A549 cells. S-phase cells were detected with BrdU staining, and mitotic cells were stained with hosphor-Histone H3 antibody. Cell images were acquired using the cellomics HCS Reader with a 10X objective lens.

From Figure 4 and Figure 5, panduratin A treatment resulted in G2/M phase arrest in A549 cells. BrdU and hosphor-histone H3 stained nuclear DNA content to distinguish 2N (G1 phase) and 4N (G2/M phase). The apoptosis induced by panduratin A is presumably a consequence of G2/M, with cells mostly being stained with mitosis-specific hosphor-histone H3 rather than BrdU.

#### 2.1.5. Cytotoxicity Assay

**Figure 6:**
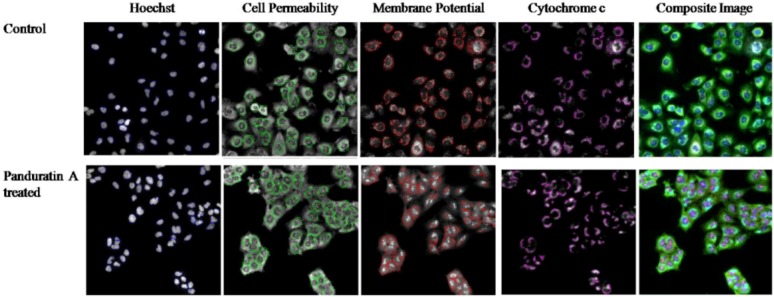
Representative images of A549 cells treated with medium alone and 5 µg/mL of panduratin A, all at 20X, and stained with Hoechst for nuclear, cell permeability dye, mitochondrial membrane potential dye and cytochrome c. The images from each row are obtained from the same field of the same treatment sample. A549 produced a marked reduction in nuclear area, mitochondrial membrane potential, and marked increases in membrane permeability. Circular outlines indicate the areas within cells in which fluorescence intensity is measured.

**Figure 7:**
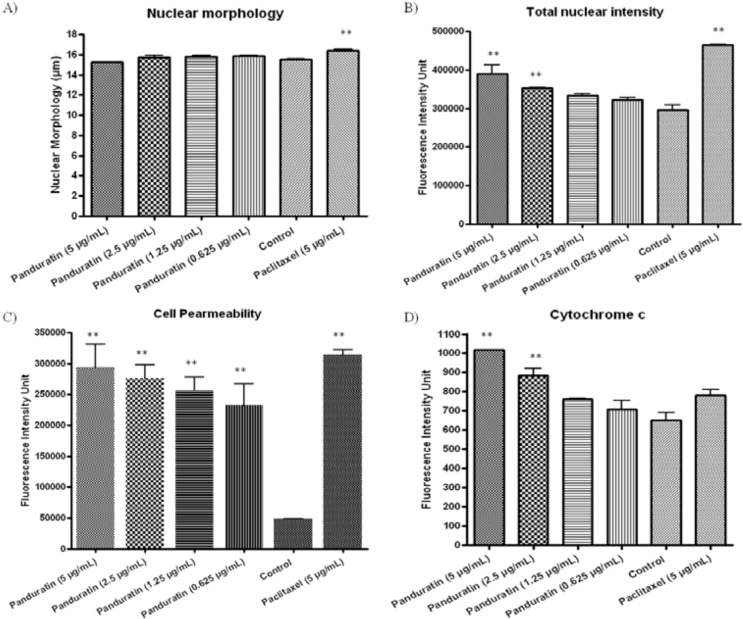
Changes in **A)** nuclear size and morphology, **B)** DNA content, **C)** cell permeability and **D)** cytochrome c localization were all measured simultaneously in A549 cells. Following treatment with panduratin A, we saw statistically significant cell loss (data not shown), nuclear condensation, increased total nuclear intensity, increased cell permeability, and cytochrome c release from mitochondria with good p values.

This assay enables simultaneous measurement of several cell-health parameters: nuclear morphology, DNA content, cell membrane permeability and cytochrome c localization. Typical cytotoxic changes are illustrated in Figure 6 and Figure 7. Panduratin A induced decreases in cell number (data not shown); nuclear area and mitochondrial membrane potential are readily visible, as are the increases in plasma membrane permeability. These effects occurred more rapidly, and followed a dose-response pattern. Cytotoxic effects were considered to occur only when the rate of change of fluorescence was unmistakably greater than for the negative controls.

#### 2.1.6. Apoptosis Determination

**Figure 8:**
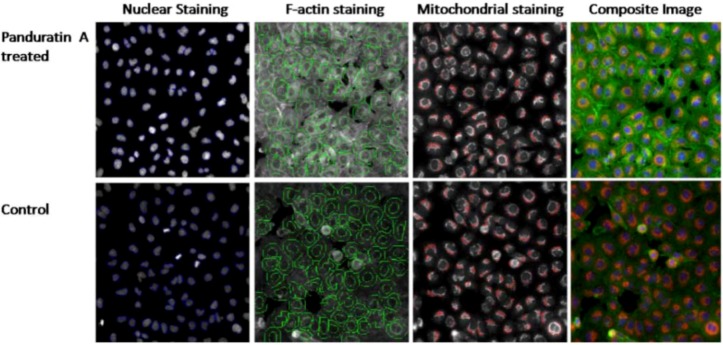
Fluorescence photomicrographs of panduratin A-induced changes in apoptosis parameters *acquired from the ArrayScan HCS Reader*. Effects of incubation of A549 cells with panduratin A, compared to controls, respectively, on nuclear area, mitochondrial membrane potential and F-actin. Circular outlines indicate the areas within cells in which fluorescence intensity is measured.

**Figure 9:**
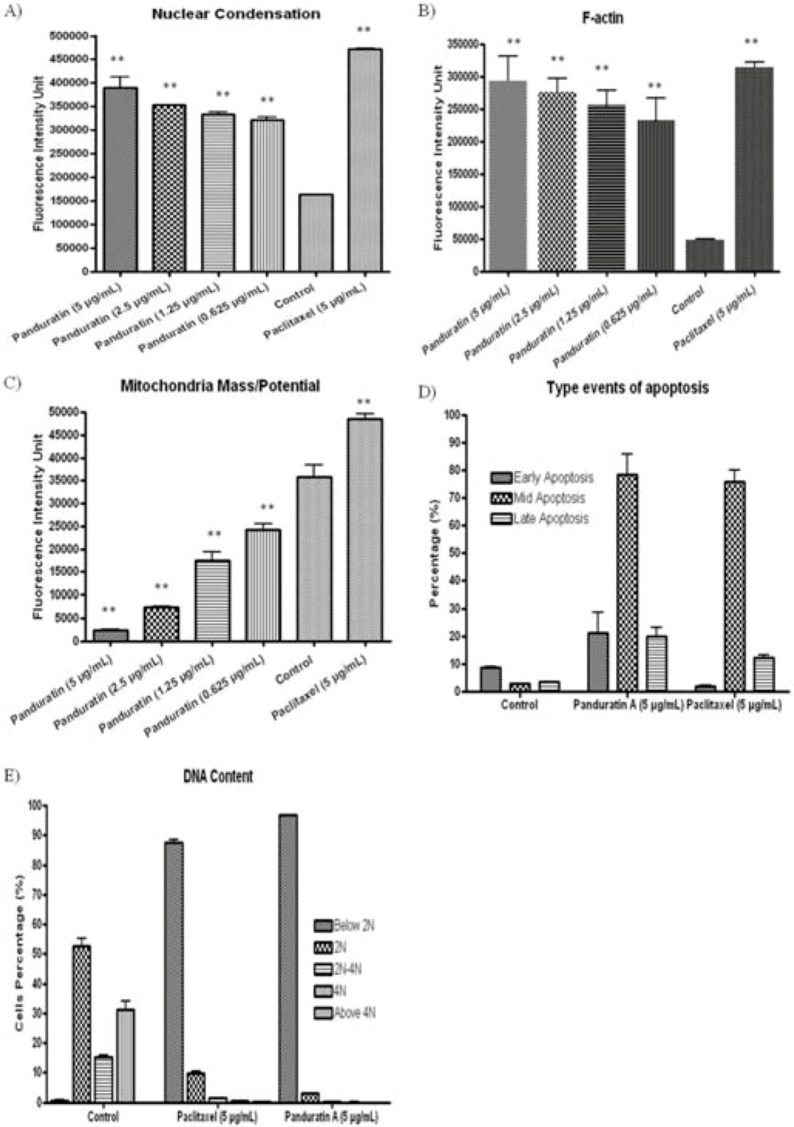
Quantitative analysis of panduratin A mediated. Dose-response histogram for each apoptosis parameter. Panduratin A increased the percentage of cells with condensed chromatin and F-action content in a concentration-dependent fashion at the same time caused loss of mitochondrial mass/potential.

To confirm the apoptosis induced by panduratin A, the changes in nuclear condensation, f-actin contents and mitochondrial mass/potential were determined simultaneously after treatment with different concentrations of panduratin A for 24 hours. Panduratin A induced dose-dependent increase of fluorescence intensity of nuclei (Figure 8 and Figure 9A), f-actin content (Figure 8 and Figure 9B) and mitochondrial mass/pontential (Figure 8 and Figure 9C) of A549 cells.

Image-based cell analysis can also provide information on cell density and thus give information about proliferation in the cell culture. The method described is not limited to studies of the three parameters presented here. By altering the selection of probes, we can study the activity of different enzymes or organelles [9]. Based on the parameter that been measured, we can define the stages of apoptosis (early, mid and late) upon treatment with 5 µg/mL of panduratin A. From Figure 9D, 70% of the A549 cells treated with panduratin A were at the mid stage of apoptosis. It would also be possible to extract information about the cell cycle phase for each cell based on the total intensity of the Hoechst 33342 or DAPI staining [10]. From Figure 9E, based on the DNA content in the cells after treatment with panduratin A, we can separate the <2N, 2N, 2N-4N, 4N and >4N of cell cycle phase.

#### 2.1.7. NF-kB Activity

**Figure 10:**
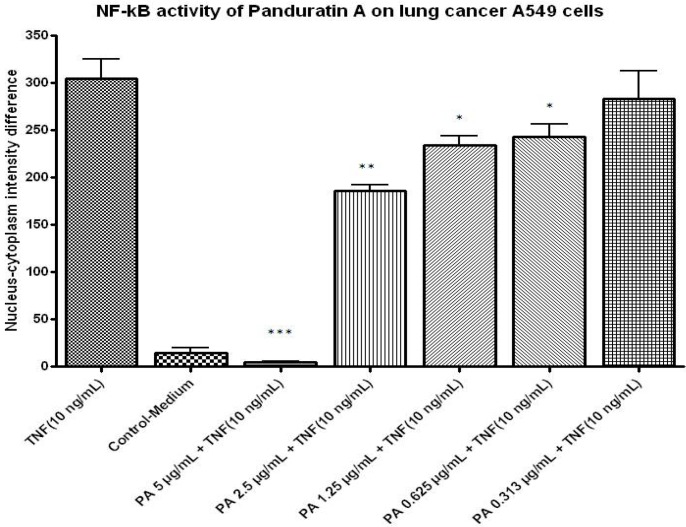
Dose-response histogram of panduration A treated A549 cells for 2 hours and then stimulated for 30 minutes with 10 ng/mL TNF-α for quantitative image analysis of intracellular targets.

**Figure 11:**
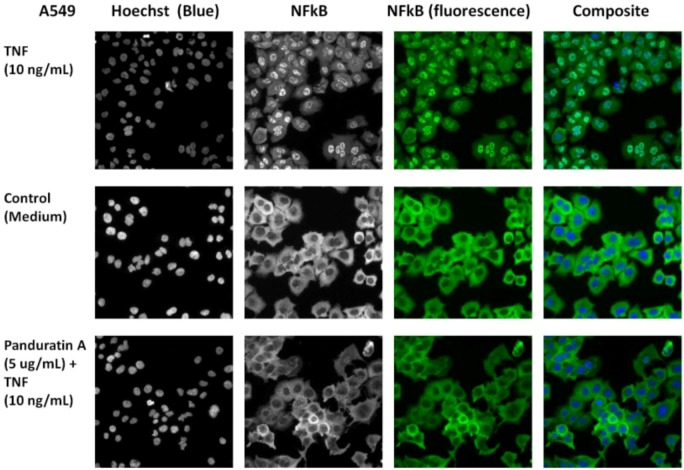
Stained A549 cells were treated with panduration A (5 µg/mL) for 2 hours and then stimulated for 30 minutes with 10 ng/mL TNF-α (NF-κB activation).

In this study, we tested panduratin A for its *in vitro* inhibitory effects against NF-κB translocation activated by TNF-α and illustrated by HCS assay. Panduratin A exhibited significant inhibitory effects on the activation of NF-κB (Figure 10). In parallel, the morphological changes of NF-κB translocation indicated by immunofluorescence staining (Figure 11) showed an inhibitory effect of panduratin A on TNF-α-induced NF-κB translocation in a dose-dependent manner. In cells treated with control, most of the fluorescence staining for NF-κB are in the cytoplasm and rare NF-κB staining in nuclei area. When stimulating cells with the TNF-α alone, NF-κB staining significantly increased in nuclei area, suggesting that NF-κB translocated from cytoplasm into the nucleus. However, A549 cells were treated with 5 μg/mL of panduratin A, NF-κB translocation induced by TNF-α was inhibited.

### 2.2. Discussions

Several plant-derived bioactive agents have been shown to induce apoptosis in a number of experimental models of carcinogenesis [11]. Panduratin A had been shown to induce apoptosis in HT-29, CaP and DU145 [3]. In the present study, we investigated the anti-proliferative activity of panduratin A and the underlying mechanism in A549 cells with the current advanced technology of HCS and RTCA.

In this study, panduratin A at higher concentration initially induces a cytotoxic effect which is mainly due to cell death. Remarkably, the cells recover from the initial cytotoxic effect of the drug and start to re-proliferate at 3.13 and 6.25 µg/mL after 40 hours of treatment. While it remains to be determined if this phenomenon is due to metabolism and inactivation of panduratin A or due to the emergence of a panduratin A-resistant subpopulation, this experiment clearly exemplifies the tremendous advantage of the real-time measurements offered by the RTCA system and allows us, the user, the opportunity to observe and assess the entire history of drug interaction with the target cells which provides further information in addition to cell viability or cytotoxicity. The phenomenon observed would have been easily missed by traditional single- or end-point assays such as MTT assay. On the hand, there was evidence that panduratin A treatment had no to little effect on normal human epithelial and fibroblast cells [4], hencer it’s suggested that panduratin A has selective cytotoxicity towards cancer cells.

Induction of apoptosis and/or inhibition of cell proliferation are highly correlated with the activation of a variety of intracellular signaling pathways leading to arrest the cell cycle in the G1, S, or G2/M phase of the cell cycle [12]. Cell cycle regulation and its modulation by various plant-derived agents have gained widespread attention in recent years [13]. With paclitaxel, cells are only seen in G2/M-blocked [14]. The apoptotic pathway for paclitaxel has been directly examined [14]. Nocodazole, which induces M phase arrest, is frequently used for a G2/M checkpoint assay besides paclitaxel [15]. In the present study, apoptosis induced panduratin A is presumably a consequence of either G2/M block or the DNA damage due to abnormal mitotic arrest. We need to further investigate the effect of panduratin A on the expression of G2/M regulatory proteins like cdc family.

Screening potential drugs for cytotoxicity is an essential aspect of the drug discovery process since cytotoxicity is a complex process affecting multiple parameters and pathways. After toxic insult, cells often undergo either apoptosis or necrosis. In this study, panduratin A treatment of A549 epithelial lung carcinoma cells led to a remarkable induction of apoptotic cells. Early during the initiation of apoptosis, cells lose contact with neighboring cells. Membranes and organelles including mitochondria are well preserved during early apoptotic cell death. Morphological hallmarks of apoptosis in the nucleus are chromatin condensation and nuclear fragmentation. Condensation and fragmentation of the nucleus can be seen. Panduratin A induced decreases in cell number, nuclear area, cell morphology, cell membrane permeability, cytochrome c localization and mitochondrial membrane potential are readily visible, as are the increases in DNA content and plasma membrane permeability.

In addition to the nuclear fragmentation, the induction of cell death increases the F-actin content. Changes in the actin cytoskeleton have been reported as a potential parameter related to apoptotic changes [16]. However, this correlation is not observed in all cases. The actin cytoskeleton is known to be crucial in mediating cell responses to both internal and external signals. The exact role for actin in the panduratin A-induced cell death pathway remains unclear but there is now a significant amount of circumstantial evidence that indicates a role for actin in triggering cell death. Some of the clearest results supporting links between actin and apoptosis pathways in mammalian cells come from studies using drugs that affect actin turnover [16].

Besides that, a reduction in mitochondrial membrane potential could be seen after 24 hours of treatment with panduratin A. Mitochondria release apoptogenic factors through the outer membrane and dissipate the electrochemical gradient of the inner membrane. This is thought to occur via formation of the mitochondria permeability transition. Mitochondria have been described to play a key role and perhaps even a central role in the apoptotic process [17] due in part to the mitochondria being the ‘junction’ of at least two distinct signaling pathways. Hence, mitochondrial dysfunction has been shown to participate in the induction of apoptosis. In some apoptotic systems, loss of mitochondrial membrane potential (Δψm) may be an early event in the apoptotic process. However, there are emerging data suggesting that, depending on the model of apoptosis, the loss of Δψm may not be an early requirement for apoptosis, but on the contrary may be a consequence of the apoptotic-signaling pathway [17]. Mitochondrial control of apoptosis has been described at several levels: [6] maintenance of ATP production and [18] Δψm and mitochondrial membrane permeability for the release of certain apoptogenic factors from the intermembrane space into the cytosol [19]. Cytochrome c release has been suggested associated mitochondrial swelling and thus ruptures of the outer mitochondrial membrane [20]. Thus, in some systems, dissipation of Δψm has been associated with cytochrome c release from the mitochondria [21].

In this study, we tested panduratin A for its *in vitro* inhibitory effects against NF-κB translocation from cytoplasm to nucleus activated by TNF-α. Panduratin A exhibited significant inhibitory effects on the activation of NF-κB. This result provided a new evidence to support the suggestion that the apoptosis effect of panduratin A may occur through mechanisms of NF-κB inhibition. Aberrant regulation of NF-κB pathway has been shown in most of human cancers, including both solid tumors and hematological malignancies [22]. It has been shown that inhibition of the NF-κB signaling pathway could reduce cancer cells growth and induce apoptosis of cancer cells [23]. The activation of this signaling pathway could be able to promote tumor cells proliferation, angiogenesis, invasion, metastasis and block apoptosis [7]. Recently, NF-κB inhibitors have emerged as new therapeutic targets for cancer disease. Further studies should be carried out to investigate the mechanism of inhibitory action.

Taken together, this study provided the first evidence that panduratin A inhibited A549 cell proliferation by apoptosis, which was associated with inhibition of translocation of NF-κB from cytoplasm to nucleus and G2/M phase arrest. These abilities of panduratin A to induce apoptosis and cell cycle arrest implies its potential as a chemotherapeutic agent or NF-κB inhibitor because many anticancer drugs are known to achieve their anticancer function by inducing apoptosis and cell cycle arrest in tumor cells. To the best of our knowledge, this was the first high content screening and real time viability assay conducted on panduratin A. Although the precise molecular mechanism by which apoptosis is induced by panduratin A remains unclear, it might be a potential anticancer agent against A549.

## 3. Experimental

### 3.1. Materials

All solvents (HPLC grade) were purchased from Fisher Scientific. A549 non-small cell lung cancer cell line was purchased from ATCC (Rockville, MD, USA). RPMI medium, penicillin, streptomycin solution and 0.25% trypsin solution were purchased from Invitrogen (Rockville, MD, USA). MTT, DMSO and heat-inactivated fetal bovine serum and paclitaxel were purchased from Sigma-Aldrich Chemicals (Saint Louis, MO, USA). Cell culture treated 96-well plates and cell culture flasks were purchased from Orange Scientific (Braine-l'Alleud, Belgium).

### 3.2. Plant Materials and Extraction

*B. rotunda* (4 g) and a voucher specimen with assession No. KU0098 is kept in the Phytochemistry Herbarium, University of Malaya. Dried plant materials were extracted twice with methanol (50 mL) for 48 hours at room temperature, replacing approximately the same volume of fresh methanol after the first 24 hours. The extracts were filtered through polyvinypyrrolidone to remove tannins before combining and drying *in vacuo* to obtain the crude extract (0.4 g).

### 3.3. Fractionation and Preparation of Compounds

The crude extract was first fractionated using reversed phase C_18_ analytical HPLC/LCMS-IT-TOF on a Shimadzu instrument equipped with a PDA detector and 80:20 splitting to a microtiter plate fraction collector (Gilson FC204). The column used was a Waters Xbridge RPC_18_ column (2.1 x 50 mm, particle size 2.5 μm), using acetonitrile and water as the mobile phase and a gradient of 10 to 100% (v/v) over 7 minutes, flow rate of 0.5 mL/min and column temperature of 40 °C. Fractions were collected every 0.5 minutes from run start until 9 minutes into a 96-well microtitre plate. The solvents were removed and the fractions were then re-dissolved in appropriate assay buffer to give a concentration equivalent to the IC_50_ concentration of the crude extract for cytotoxicity testing.

Biological assay of the collected fractions located the cytotoxicity in the 6-7 mins region. This region contained a single major peak that was identified as panduratin A based on MS/MS data. Panduratin A was subsequently isolated from crude extract by preparative reversed-phase HPLC (Waters Nova-Pak C_18_ column, particle size 6 μm, 25 x 100 mm) using acetonitrile and water as eluents. A gradient of 60% to 100% (v/v) acetonitrile in water at a flow rate of 12 mL/min was applied over 50 minutes. Identity and purity (>98%) of the isolated panduratin A were determined by analytical HPLC and nuclear magnetic resonance (^1^H-NMR) spectroscopy. The ^1^H-NMR in CDCl_3_ was found to be identical to that previously reported [24].

### 3.4. Cell Culture

A549 cell line used in this study was maintained at 37 °C incubator with 5% CO_2_ saturation. Cells were cultured in RPMI media containing 10% FBS and 1% penicillin and streptomycin.

### 3.5. Real-Time Cell Proliferation Assays

In vitro cell proliferation was assessed in xCELLigence Real-Time Cellular Analysis (RTCA) system (Roche, Germany). Briefly, background measurements were took after adding 50 µL of the appropriate medium to the wells of the 16X E-plate. Cell suspension (1.25 × 10^4^ cells/well) was added to the wells. The attachment and proliferation of the cells were monitored every 5 minutes using RTCA system. Approximately 18 hours after seeding, when the cells in the log growth phase, the cells were treated with 100 µL of panduratin A in various concentrations (µg/mL) dissolved in cell culture media and continuously monitored for up to 72 hours. The cells were also treated with medium alone, which served as vehicle control. Cell sensor impedance was expressed as an arbitrary unit called the Cell Index.

### 3.6. MTT Assay

Assays were performed after 24, 48 and 72 hours treatment periods. 50 µL of MTT solution (2 mg/ml) was transferred to each well. Plates were incubated for 2 hours at 37 °C and discarded the supernatants. DMSO was added to ensure total solubility of formazan crystals and absorbance was recorded at 570 nm with Plate Chameleon V microplate reader (Hidex, Turku, Finland). The percent viability was expressed as absorbance in the presence of test compound as a percentage of that in the vehicle control.

### 3.7. Cell Cycle Phases

The Cellomics Cell Cycle Kit is for quantification of nuclear DNA content to distinguish DNA replication in S phase cells and mitosis marker in M phase cells. This kit allows direct measurements of bromodeoxyuridine (BrdU) incorporation and mitosis-specific histone H3 phosphorylation. The assay was performed according to manufacturer’s instructions. Plates were analyzed using the ArrayScan high content screening (HCS) system (Cellomics Inc., Pittsburgh, PA, USA).

### 3.8. Multiparametric Cytotoxicity Assay

Multiparametric Cytotoxicity HCS assays were used as described in detail previously by [25]. Plates were analyzed using the ArrayScan HCS system.

### 3.9. Apoptosis Determination

Multiparameter Apoptosis Kit provides quantitative analysis of apoptosis by analysis of nuclear morphology, mitochondrial mass/potential and F-actin content. Apoptosis HitKit^TM^ reagents (Cellomics) was used according to the manufacturer’s instructions. Plate was evaluated on the ArrayScan HCS Reader and analyzed with Apoptosis BioApplication software.

### 3.10. Detection of NF-kB Activity

HCS was used to measure the inhibitory effects of panduratin A on TNF-α-induced NF-κB activation, *i.e.* nuclear translocation of NF-κB. The experiments were performed according to manufacturer’s instructions for the NF-κB activation kit (Cellomics). ArrayScan reader was used to quantify the difference between the intensity of nuclear and cytoplasmic NF-κB-associated fluorescence, reported as translocation parameter.

### 3.11. Image Acquisition and Cytometric Analysis

Plates with stained cells were analyzed using the ArrayScan HCS system (Cellomics). This system is a computerized automated fluorescence imaging microscope that automatically identifies stained cells and reports the intensity and distribution of fluorescence in individual cells. The Array-Scan HCS system scans multiple fields in individual wells to acquire and analyze images of single cells according to defined algorithms. In each well, 1,000 cells were analyzed. Automatic focusing was performed in the nuclear channel to ensure focusing regardless of staining intensities in the other channels. Images were acquired for each fluorescence channel, using suitable filters. Images and data regarding intensity and texture of the fluorescence within each cell, as well as the average fluorescence of the cell population within the well were stored in a Microsoft SQL database for easy retrieval. Data were captured, extracted and analyzed with ArrayScan II Data Acquisition and Data Viewer version 3.0 (Cellomics).

### 3.12. Statistical Analysis

Each experiment was performed at least two times. Results are expressed as the means value ± standard deviation (SD). Statistical analysis was performed with one-way analysis of variance (ANOVA), with Dunnett's Multiple Comparison Test to identify between-group differences using GraphPad Prism software (version 4.0; GraphPad Software Inc., San Diego, CA). Statistical significance is expressed as ***, P < 0.001; **, P < 0.01; *, P < 0.05. Log IC_50_ calculations were performed using the built-in algorithms for dose-response curves with variable slope. A fixed maximum value of the dose-response curve was set to the maximum obtained value for each drug.

## 4. Conclusions

We have presented a real-time cytotoxicty mediated by panduratin A and a series of multiparametric HCS assays demonstrating multiple apoptotic parameters on A549 adherent cells. Panduratin A has benefits as a NF-κB inhibitor to inhibit the proliferation of cancer cells by blocking cells in G2/M phase and by inducing apoptosis. Further studies should be carried out to investigate the mechanism of inhibitory action, such as quantitative analysis of IκB kinase activity, IκB alpha phosphorylation and degradation, p50 and p65 nuclear translocation, DNA binding and NF-κB-dependent reporter gene expression.
